# The Status of Institutional Dispensers

**Published:** 1914-04-11

**Authors:** 


### THE STATUS OF INSTITUTIONAL DISPENSERS.

The Bill which has been introduced in the House of Commons to set up a qualification for assistant compounders of medicine has now been circulated to members. The main object of the Bill was recently stated in The Hospital, but the provisions are rather more comprehensive than had been supposed. The first clause provides that from and after January 1, 1916, it shall be unlawful for any person "not being a duly registered chemist and druggist or registered under this Act" to act as assistant to a chemist in compounding any medicine prescribed by a duly qualified medical practitioner; exceptions, however, are made in favour of qualified medical practitioners and registered pharmaceutical apprentices provided the latter are supervised by registered chemists. The second clause empowers the Pharmaceutical Society to make by-laws providing for the examination and registration of assistants. Clause 3 provides for the registration as assistant without examination of any person who produces evidence that at the date of the passing of the Bill he was "a qualified military dispenser, or a certified assistant to an apothecary under the Apothecaries Act, 1815, or had been for a period of not less than three years immediately preceding that date actually engaged and employed by a duly qualified medical practitioner or by a chemist and druggist as an assistant in compounding medicines." It is further provided in Clause 5 that a qualified military dispenser or a certified assistant to an apothecary shall be entitled to be registered as an assistant without examination if he satisfies the Council of the Pharmaceutical Society that he is "a person of sufficient skill and experience in compounding medicines."

It will be observed that the measure seeks to introduce into English pharmacy law a new principle, since at present it is not unlawful for an unqualified person to compound medicines. The new principle is undoubtedly a sound one, and its adoption by the Legislature would provide another safeguard for the public and at the same time give a status to dispensers who, though capable and experienced, have hitherto been denied statutory recognition. There appears, however, to be nothing in the Bill to regulate the dispensing of medicines in those public institutions in which a qualified chemist is not employed. Although this omission is not a serious one from the point of view of the larger hospitals both in London and the provinces, where properly qualified persons are employed, there are, as has been pointed out in The Hospital on previous occasions, a number of institutions in which the compounding of medicines is not in the hands of qualified persons. There will, however, be ample opportunities before the Bill reaches its final stage to consider the desirability of introducing amendments with the object of making provision for the dispensing of medicines in other places than chemists' shops. As to the classes of persons who would be entitled to registration without examination, it is probable that the proposals are sufficiently wide to include most of the trained dispensers in public institutions, although they would not entitle to registration persons who though inexperienced occasionally perform the duties of a dispenser. It would obviously be contrary to public policy to admit to the register persons who have had no real and continuous experience, and it is to be hoped that whatever amendments may be made to make clearer the position of institutional dispensers who apply for registration, no loophole will be left for those who are obviously without skill and experience. The Bill was down for second reading on Friday last week, but the application of the five o'clock rule prevented the motion from being put, and there seems little likelihood that the Bill will make much progress this session.

